# Clinical Features and Endovascular Management of Iliac Arteriovenous Fistulas: A 10-Year Single Center Experience

**DOI:** 10.3389/fsurg.2022.873665

**Published:** 2022-04-14

**Authors:** Lei Ji, Guangchao Gu, Zhili Liu, Yuexin Chen, Wei Ye, Bao Liu, Changwei Liu, Yuehong Zheng

**Affiliations:** ^1^Department of Vascular Surgery, Peking Union Medical College Hospital, Chinese Academy of Medical Sciences and Peking Union Medical College, Beijing, China; ^2^Department of Vascular Surgery, School of Medicine, The First Affiliated Hospital, Zhejiang University, Hangzhou, China

**Keywords:** iliac arteriovenous fistulas, clinical features, embolization, endovascular procedures, clinical success, technical success

## Abstract

**Objective:**

Iliac arteriovenous fistulas (IAVFs) are rare abnormalities with limited reported cases. This study aimed to summarize the clinical features and experiences on the diagnosis and endovascular treatment of IAVFs.

**Methods:**

A single-center retrospective study of IAVFs from 2010 to 2019 was performed. Data including demographics, clinical characteristics, radiological images, surgical details, and follow-up were collected.

**Results:**

A total of 16 patients diagnosed with IAVFs were identified. The female to male ratio was 3:1. The mean age was 47.7 ± 10.4 years (range: 35–73 years). Leg swelling and cardiac insufficiency, especially heart failure, were the most common primary symptoms in this series, which were revealed in 68.8 and 37.5% of patients, respectively. Iatrogenic, traumatic, and congenital IAVFs were diagnosed in 62.5, 12.5, and 25.0% of patients, respectively, among which hysterectomy was thought to be the main cause for female iatrogenic IAVFs (31.3%). Anatomic analysis found that internal iliac vessels were the predilected sites of IAVFs. All the patients were treated by endovascular procedures composed of transarterial embolization (50.0%), transarterial and stage II transvenous embolization (31.3%), stage I transarterial and transvenous embolization (12.5%), and transarterial embolization plus stent implantation (6.3%). The clinical success rate was 93.8%. Minor complications including fever (18.8%) and exudation at the puncture point (6.25%) were noted and well-treated. During a follow-up period of 51.3 ± 41.9 months after operations, only one patient experienced re-emergence of heart failure because of recurrence of leiomyosarcoma; other patients recovered uneventfully with symptoms relief and no severe embolization-related complications was encountered.

**Conclusion:**

IAVFs are rare disorders and correct diagnosis requires careful history taking and physical examination, combined with proper imaging investigation. The primary goal for treatment of IAVFs was to relieve associated symptoms. Based on the results of this study, endovascular approach is safe and effective for treatment of IAVFs.

## Introduction

Iliac arteriovenous fistulas (IAVFs) are uncommon entities defined as direct communications between iliac arteries and veins, which account for 0.4–1.4% of all the AVFs (1, 2). IAVFs can be either congenital or secondary to trauma and surgery, with commonly progressive hemodynamic disturbances leading to high output cardiac insufficiency, leg swelling or ischemia, and aneurysm or vein aneurysm formation (3–5). The initial diagnosis of IAVFs is challenging because of the atypical symptoms and rarity of the presentations. Currently, there is no consensus regarding the optimal therapeutic strategy of IAVFs. Open surgery was the first-line treatment for IAVFs in the past decades, but may bring about a high mortality and morbidity (6). With the development of interventional devices and techniques, successful applications of endovascular approaches on IAVFs have been reported (5, 7).

Due to the rarity, most published studies focusing on IAVFs were case reports. In this study, we presented a relatively larger number of patients with IAVFs from a single center, with the attempt to summarize the clinical characteristics of IAVFs and to evaluate the outcome of endovascular approaches in the management of IAVFs, in order to increase our understanding of this rare disease.

## Methods

### Patients

**Figure 1:**
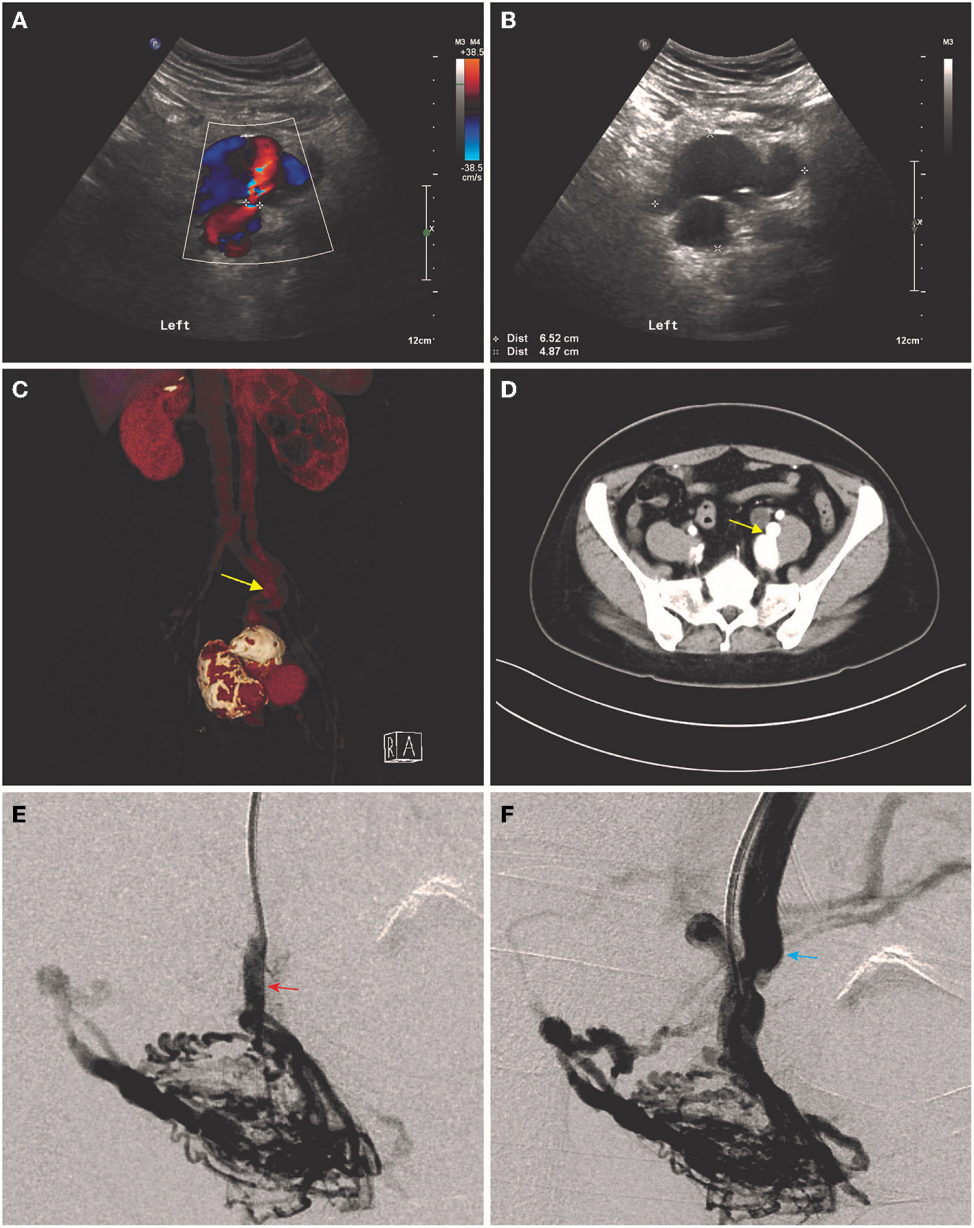
Typical imaging presentations of iliac arteriovenous fistulas (IAVFs). **(A)** Multicolored blood flow can be seen in the proximal artery during both the systole and diastole in ultrasound. **(B)** Aneurysm and venous aneurysm formation were shown in ultrasound. **(C,D)** CT angiography (CTA) and cross-sectional image showed a direct connection (yellow arrows) between internal left iliac artery and vein accompanied with aneurysm and venous aneurysm formation. **(E,F)**. Digital subtraction angiography (DSA) showed multiple connections between a branch of inflow left iliac artery (red arrow) and outflow left internal iliac vein.

This study was approved by the Ethics Committee of Peking Union Medical College Hospital (PUMCH) and a written informed consent was obtained from each patient according to the Declaration of Helsinki. From March 2010 to November 2019, a total of 16 patients were diagnosed with IAVFs and treated in the PUMCH. The demographics, clinical characteristics, radiological images, surgical details, and follow-up data were retrospectively collected. IAVFs were defined as having involvement of iliac vessels by both the inflow and outflow tracts of the AVFs. The disease was diagnosed by ultrasound (US) (Figures 1A,B) or CT angiography (CTA) (Figures 1C,D) and finally confirmed by digital subtraction angiography (DSA) (Figures 1E,F). The etiology for IAVFs was determined based on medical history, wherein acquired IAVFs were defined as fistulas formation secondary to trauma (traumatic IAVFs) or previous operation (iatrogenic IAVFs); Congenital IAVFs were defined as spontaneous formation of IAVFs without any history of trauma, surgery, or deep vein thrombosis (DVT) (8–11).

### Surgery

Fifteen patients were performed endovascular embolization under local anesthesia, including transarterial and/or transvenous embolization, and one patient was performed endovascular embolization combined with stent placement under general anesthesia. Transarterial embolization was performed following a standard procedure: Briefly, the contralateral femoral artery access was established with a 6-F, 20-cm long or 40-cm long crossover sheath, through either a groin incision or a direct percutaneous puncture. Systemic heparinization (50 U/kg) was administered intravenously. Iliac arteriography was performed to classify the presence of IAVFs as well as the feeding arteries and draining veins. Target feeding arteries were selected via a 5-F Vert catheter from the contralateral side and the coils (Interlock, Boston Scientific Corporation, USA) were injected to embolize the major target feeding arteries. For complex IAVFs with multiple major feeding vessels, multiple repetitive embolization would be planned to avoid severe surgical complications associated with prolonged surgical time and excessive use of contrast agent and to reserve enough arterial accesses for subsequent embolization, which is very likely to be needed due to the high probability of relapsing or deterioration of such complex IAVFs following single embolization. After transarterial embolization, angiography was performed to evaluate the effect of embolization. No transvenous embolization was needed, if satisfying embolization was obtained as revealed by obvious reduction of the volume and blood flow of the fistula. If significant blood flow was observed after embolizing all the major feeding arteries or the fistula had a very large size, a transvenous embolization will be performed either at the same stage or at a second stage, depending on the intraoperative situation and the patients' tolerance to the surgery, to obtain optimal embolization.

Transvenous embolization was carried out following a similar procedure to transarterial embolization; briefly, contralateral femoral vein access was established and iliac venography was performed to clarify the major draining veins of the IAVFs and then embolization agents (coils alone, coils plus gelatin sponge particles, or lauromacrogol) were injected into the major draining branches or directly into the internal iliac veins.

Procedure of stent placement was as follows: Under fluoroscopy, a guide wire and catheter were placed into the abdominal aorta. Angiography demonstrated the fistula connecting the right common iliac artery and the left common iliac vein. Coils were placed at the beginning of the right internal iliac artery. Then, an abdominal aorta covered stent was placed, followed by the release of bilateral iliac branches. The proximal end of right iliac branch was connected with the main body stent and the distal end was located in the right external iliac artery.

The technical success of the operation was defined as complete or partial occlusion of connections between iliac arteries and veins after embolization. The clinical success was defined as the relief of related symptoms, mainly heart failure and leg swelling.

### Follow-Up

The follow-up information came from outpatient or telephone review at postoperative months 1, 3, 6, and 12 and then yearly thereafter. The primary endpoint of follow-up was recurrence, while the secondary endpoint was death from complications. The recurrence was defined as reoccurring or worsening of associated symptoms. Follow-up time was censored, if the patient was lost to follow-up or died from other causes.

### Data Analysis

Categorical variables were presented as numbers and percentages and continuous variables were presented as mean ± SD values or median and interquartile range (IQR). All the statistical analyses were performed using GraphPad Prism version 7.0 (GraphPad Software Incorporation, San Diego, California, USA).

## Results

### Clinical Characteristics

A total of 16 patients diagnosed with IAVFs were identified and included in this study. Demographics and clinical characteristics were shown in Table 1. The female to male ratio was 3:1. The mean age of all the patients was 47.7 ± 10.4 years (range: 35–73 years). Regarding clinical presentations, cardiac insufficiency, as revealed by symptoms such as shortness of breath or dyspnea, was present in 37.5% of patients, leg swelling was present in 68.8% of patients, and hepatomegaly and ascites was present in only 6.3% of patients due to visceral congestion. 25.0% of patients presented with intermittent claudication (12.5%) or muscle twitch (12.5%) due to decreased arterial blood flow in the lower extremity. 18.8% of patients presented with urinary frequency and/or constipation due to the compression of the fistula. In addition, only one patient had concomitant DVT. No asymptomatic patient was found in this case series.

**Table 1:** Demographics and clinical characteristics of patients with iliac arteriovenous fistulas (IAVFs).

**No**.	**Sex**	**Age (years)**	**Cardiac insufficiency**	**Leg swelling**	**Leg ischemia**	**Visceral congestion**	**Compression**	**DVT**	**Surgical history**	**Traumatic history**	**Etiology**
01	F	58	Y	Y	N	N	N	N	Ligation of hemangioma, hysterectomy	N	Iatrogenic
02	F	36	N	Y	N	N	N	N	N	N	Congenital
03	M	45	N	N	Y	N	N	N	Lumbar discectomy	Sharp instrument injury	Traumatic
04	F	47	N	N	N	N	Urinary frequency	N	Cholecystectomy, hysterectomy	N	Iatrogenic
05	F	53	N	Y	Y	N	N	N	Lumbar discectomy	N	Iatrogenic
06	F	73	Y	Y	Y	N	N	N	Tubal ligation	N	Iatrogenic
07	F	40	N	Y	Y	N	N	N	Cesarean, appendectomy	N	Iatrogenic
08	F	56	N	N	N	N	Urinary frequency and constipation	N	Cesarean, laparotomy	N	Iatrogenic
09	F	49	Y	Y	N	N	N	N	Cesarean, hysterectomy, resection of the leiomyosarcoma	N	Iatrogenic
10	F	50	N	Y	N	N	N	Y	Open reduction and internal fixation	Intertrochanteric fracture	Iatrogenic
11	M	35	N	Y	N	N	N	N	N	N	Congenital
12	M	59	N	N	N	N	Urinary frequency	N	N	N	Congenital
13	F	45	Y	Y	N	Hepatomegaly and ascites	N	N	Hysterectomy	N	Iatrogenic
14	F	36	N	Y	N	N	N	N	N	Sharp instrument injury	Traumatic
15	F	44	Y	Y	N	N	N	N	Hysterectomy, resection of the leiomyosarcoma	N	Iatrogenic
16	M	37	Y	N	N	N	N	N	N	N	Congenital

*F, female; M, male; Y, yes; N, no; DVT, deep venous thrombosis*.

According to the diagnostic criteria elaborated in Methods, the number of iatrogenic, traumatic, and congenital IAVFs was confirmed in 62.5, 12.5, and 25.0% of patients, respectively. Hysterectomy, reported by 31.3% of patients, was thought to be the main cause of iatrogenic IAVFs. 75% (3 in 4 cases) male patients with IAVFs were congenital. The congenital to acquired IAVFs ratio in female patients was 1:11.

### Anatomical Features

Based on imaging data of CTA and DSA, the anatomical characteristics of the fistulas were shown in Table 2. The IAVFs involved the right iliac vessels, left iliac vessels, or both the sides in 43.8, 43.8, and 12.5% of cases, respectively. 93.8% (15/16) of inflow arteries were internal iliac arteries and the same proportion of outflow veins was internal iliac veins, which meant that internal iliac vessels were the predilection sites of IAVFs. Iliac aneurysm formation was found in 37.5% of patients and iliac venous aneurysm formation was found in 25.0% of patients, with 18.8% of patients possessing both the iliac aneurysm and venous aneurysm.

**Table 2:** Anatomical characteristics of iliac arteriovenous fistulas (IAVFs).

**NO**.	**Location**	**Inflow arteries**	**Outflow veins**	**Aneurysm**	**Venous aneurysm**
01	Right	Bilateral internal and right external iliac arteries	Right internal iliac vein	N	N
02	Right	Right internal iliac artery	Right internal iliac vein	Y	Y
03	Right	Right internal iliac artery	Right internal iliac vein	N	Y
04	Left	Left internal iliac artery	Left internal iliac vein	Y	Y
05	Bilateral	Right common iliac artery	Left common iliac vein	N	N
06	Left	Left internal iliac artery	Left internal iliac vein	N	N
07	Right	Right internal iliac artery	Right internal iliac vein	N	N
08	Right	Right internal iliac artery	Right internal iliac vein	Y	N
09	Right	Right internal iliac artery	Right internal iliac vein	N	N
10	Left	Left internal iliac artery	Left internal iliac and femoral veins	N	N
11	Left	Left internal iliac artery	Left internal iliac vein	N	N
12	Left	Left internal iliac artery	Left internal iliac vein	Y	Y
13	Bilateral	Bilateral internal iliac arteries	Right internal iliac vein	Y	N
14	Left	Left internal iliac artery	Left internal iliac vein	N	N
15	Left	Left internal iliac and superficial Femoral arteries	Left internal iliac vein	N	N
16	Right	Right internal iliac artery	Right common iliac vein	Y	N

*Y, yes; N, no*.

### Surgical Outcomes

**Figure 2:**
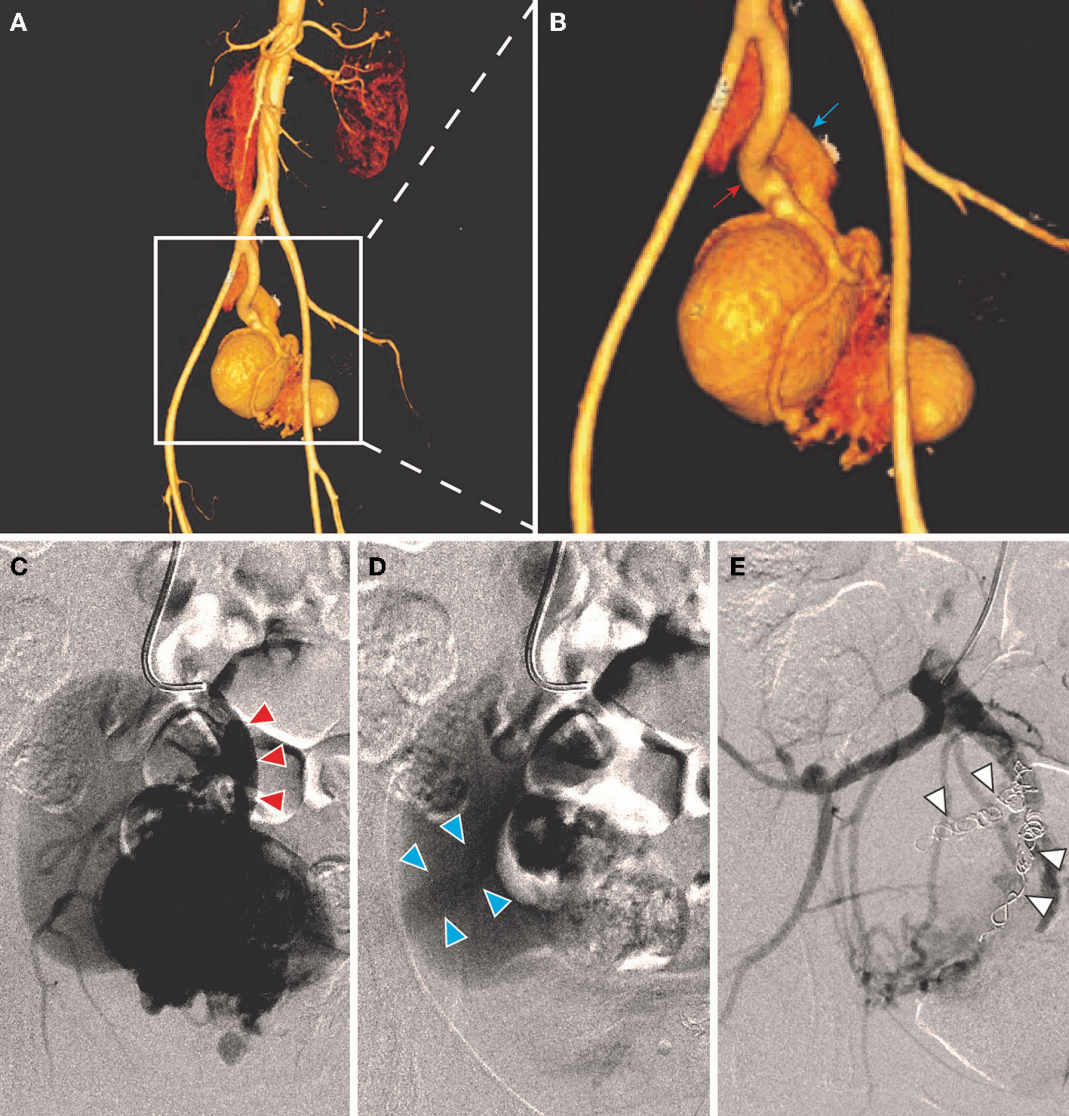
A 36-year-old female (case 2) was diagnosed as IAVFs and treated with transarterial embolization. **(A,B)** CTA showed communications between right internal artery (red arrow) and vein (blue arrows). **(C,D)** Transarterial angiography showed inflow arteries (red triangle arrows) and outflow veins (blue triangle arrows). **(E)**. Coil embolization of branches of right internal iliac artery (white triangle arrows).

**Figure 3:**
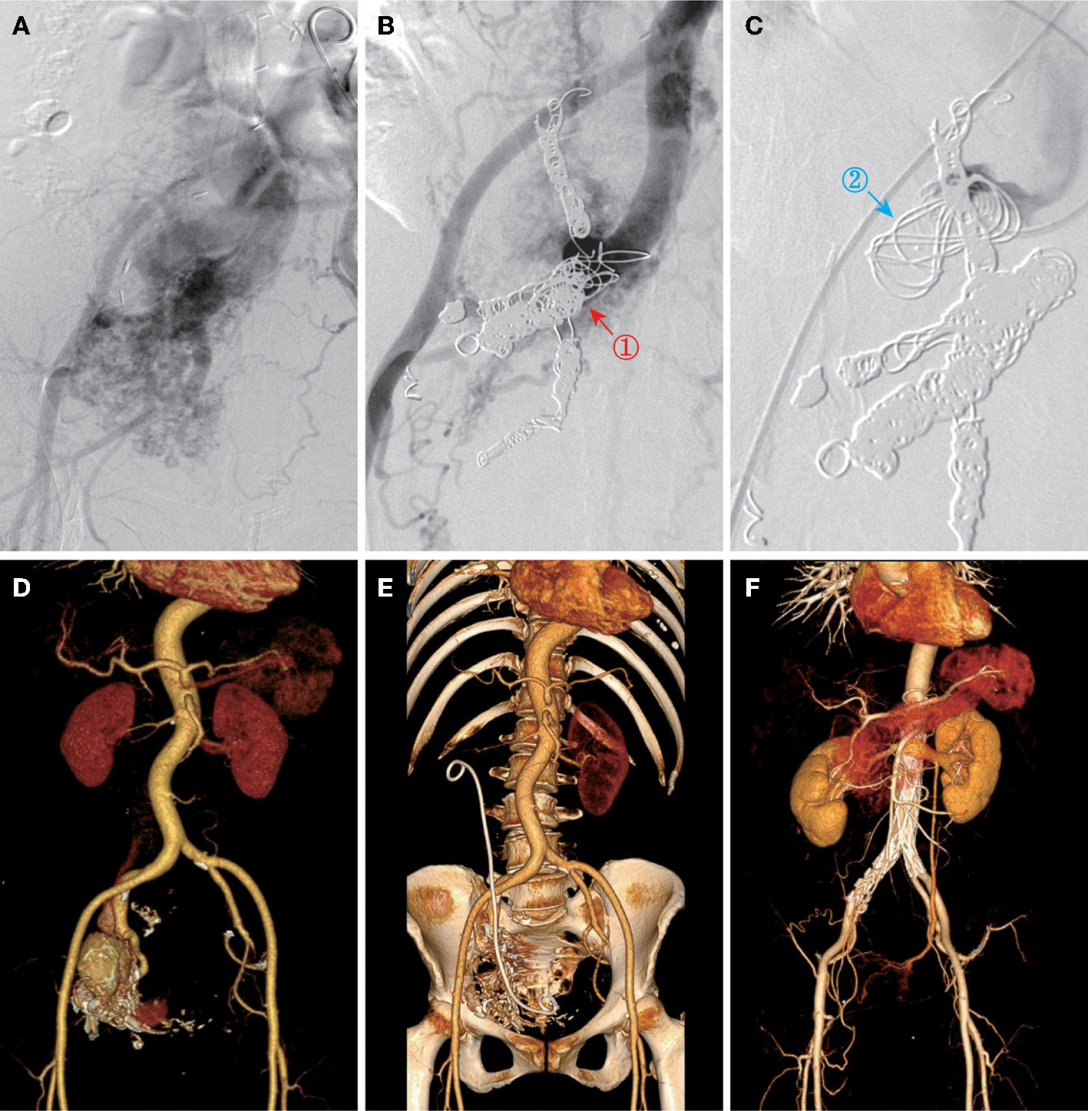
Other three types of endovascular approach. **(A)** Angiography showed IAVFs in a 49-year-old female (case 9). **(B)** Transarterial embolization of inflow right internal iliac arteries on stage I (red arrow and “”). **(C)** Transvenous embolization of outflow right internal iliac vein on stage II (blue arrow and “”). **(D,E)** Pre- and postoperative CTA images of a 56-year-old female (case 8) who was performed transarterial and transvenous embolization once time for right internal IAVFs. **(F)** Postoperative CTA image of a 53-year-old female (case 5) who was performed transarterial embolization plus stent placement for bilateral common IAVFs.

All the patients were performed endovascular therapy composed of four types of surgical procedures (Table 3): (1) transarterial embolization for 50.0% of patients (Figure 2); (2) transarterial and stage II transvenous embolization for 31.3% of patients (Figures 3A–C); (3) stage I transarterial and transvenous embolization for 12.5% of patients (Figures 3D,E); and (4) transarterial embolization plus stent implantation for 12.5% of patient (Figure 3F). 37.5% of patients were performed multiple operations as planned, due to the complexity of IAVFs, among which 18.8% of patients required 2 operations (transarterial and stage II transvenous embolization) and another 18.8% of patients required 3 operations (3 transarterial embolization for 1 case and 2 transarterial embolization and 1 stage II transvenous embolization for 2 cases). The mean number of operations of all the patients was 1.5. The materials used in surgeries were mainly coils of different diameters. The technical success rate was 100% and the overall clinical success rate was 93.8% (15/16). Only one patient presented as right heart failure and pulmonary hypertension without noticeable improvement after surgery, which might be correlated with the complex cardiac comorbidities including atrial septal defect and severe tricuspid insufficiency. 18.8% of patients using gelatin sponge particles experienced low-grade fever after surgery and returned to normal within 2 days. Puncture site exudation was found in one patient and treated well by suture of puncture point under local anesthesia. All the patients were discharged within 3 days after operation.

**Table 3:** Surgical characteristics of patients with iliac arteriovenous fistulas (IAVFs).

**NO**.	**Anesthesia**	**Surgical procedure**	**Surgical approach**	**Surgical materials**	**Number of operations**	**Technical success**	**Clinical success**	**Perioperative complications**
01	Local	Embolization	Transarterial	Coils	3	Y	N	N
02	Local	Embolization	Transarterial	Coils	1	Y	Y	N
03	Local	Embolization	Transarterial and stage II transvenous	Coils and GSPs	2	Y	Y	Fever
04	Local	Embolization	I stage transarterial and transvenous	Coils	1	Y	Y	Fever
05	General	Embolization and stent implantation	Transarterial	Coils and a covered stent	1	Y	Y	N
06	Local	Embolization	Transarterial	Coils	1	Y	Y	N
07	Local	Embolization	Transarterial and stage II transvenous	Coils and GSPs	3	Y	Y	N
08	Local	Embolization	I stage transarterial and transvenous	Coils	1	Y	Y	N
09	Local	Embolization	Transarterial and stage II transvenous	Coils	2	Y	Y	Exudation at the puncture point
10	Local	Embolization	Transarterial	Coils	1	Y	Y	N
11	Local	Embolization	Transarterial	Coils	1	Y	Y	N
12	Local	Embolization	Transarterial and stage II transvenous	Coils, GSPs and lauromacrogol	3	Y	Y	Fever
13	Local	Embolization	Transarterial and stage II transvenous	Coils	2	Y	Y	N
14	Local	Embolization	Transarterial	Coils	1	Y	Y	N
15	Local	Embolization	Transarterial	Coils	1	Y	Y	N
16	Local	Embolization	Transarterial	Coils	1	Y	Y	N

*Y, yes; N, no; GSPs, gelatin sponge particles*.

### Follow-Up

**Figure 4:**
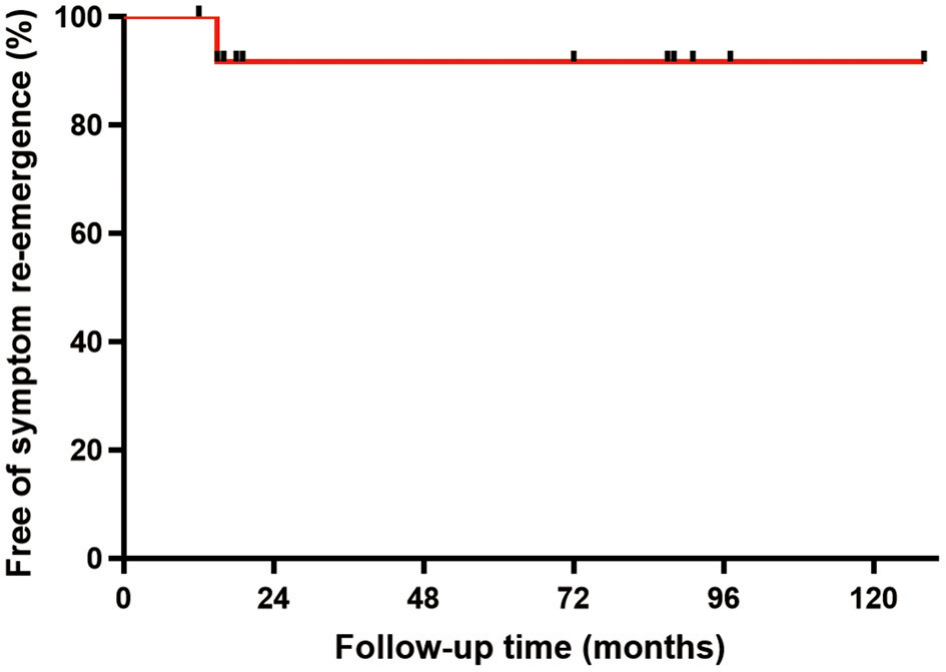
Symptom re-emergence free analysis. As shown in the survival curve, the overall no-recurrence rate was 93.3% (14/15).

No death or readmission was observed during the 30-day postoperative follow-up. Fifteen patients were successfully followed-up for an average period of 51.3 ± 41.9 months (range: 12–128 months) after surgery. All the patients recovered uneventfully with no long-term complications, mortality, or signs of recurrence, except that re-emergence of heart failure was experienced in one patient due to the recurrence of a leiomyosarcoma. The overall curve for free of symptom re-emergence was shown in Figure 4.

## Discussion

Arteriovenous fistulas can occur in various vessels, relying upon the etiology, in which IAVFs are rare and often reported as the complications of abdominal surgery or trauma. Previous studies on IAVFs described either only an isolated individual case or small case series. A systematic review including all the published cases of abdominal AVFs until September 2013 reported 48 articles with 53 patients, in which only one patient was diagnosed with IAVFs, emphasizing its rarity (12). To the best of our knowledge, we presented the clinical features and surgical outcomes of the largest series of patients with IAVFs.

IAVFs have consistent signs with typical AVFs, including pulsating mass, palpable thrill, or audible bruit and might present various types of symptoms as a consequence of hemodynamic disturbance (13). In this case series, leg swelling was the most frequent symptom in patients with IAVFs. From the anatomical standpoint, iliac veins are close to saphenous venous valve, which could directly induce venous valve insufficiency, venous hypertension, and vessel dilatation and finally result in leg swelling; part of patients simultaneously presented with varicose veins in the lower extremities. The second symptom that needs special attention is cardiac insufficiency, mainly including congestive heart failure and pulmonary hypertension, occurring in 37.5% (6/16) patients with IAVFs in this study. Previous study of spontaneous aortocaval fistulas showed that the diagnosis of 66.7% (4 in 6 cases) of patients was incidentally made during the operation (14). Severe heart failure with unknown etiology is life-threatening; therefore, an abdominal examination should be performed for a patient present with short of breath or dyspnea and initial diagnosis of heart failure (15). In addition, two patient presented intermittent claudication induced by lower limb ischemia, which was rarely reported although the pathophysiology was easy to understand (16). Taken together, the clinical symptoms of IAVFs are often confusing, reflecting the importance of physical examination.

The etiological diagnosis of IAVFs was made on the basis of reported medical history combined with literature review. The most frequent iatrogenic AVFs are reported in the femoral vessels resulting from commonly used percutaneous approach (17). In contrast, in the 10 iatrogenic patients with IAVFs, uterine surgeries, including cesarean and hysterectomy, were thought to be the causes of 70% iatrogenic IAVFs, which also explained the high ratio of female patients. Several studies have found female patients presenting with IAVFs, uterine or pelvic AVFs after hysterectomy, suggesting the quite possible relationship between hysterectomy and IAVFs (18–20). However, there is still no direct evidence that uterine surgeries could lead to IAVFs. From the perspective of anatomy, the uterus is adjacent to the iliac vessels and iliac vascular injury during hysterectomy is not uncommon. A large retrospective study from China focusing on 18,447 women with stage IA-IIB cervical cancer undergoing hysterectomy reported 0.15% of patients experienced vascular injury (21). In addition, life-threatening vascular complications such as iliac artery rupture is not uncommon during lymphadenectomy of gynecology oncology surgery, which composes the important procedures during surgical staging of ovarian cancers (22, 23). In consistent with these cases from another point, IAVFs sometimes occurred as a complication of disk surgery (2). Lumbar surgery is mostly performed on L4–L5 vertebrae and intervertebral disk (24). If IAVFs were induced, it could appear not only early but also very late after operation (25, 26). Additionally, as reported in the literatures, IAVFs can present with concomitant aneurysm or venous aneurysm, which is also dangerous for the risk of rupture (15). Aneurysm formation was due to the shear stress in the arterial wall and venous aneurysm development is likely secondary to increased inflow from the arterial portion of the AVFs, leading to venous hypertension that causes weakening and subsequent stretching of the vein wall. Incidental discovery of iliac venous aneurysm on, otherwise, routine ultrasound scanning has been previously been described; however, the described repair was performed in an open approach. Besides clinical symptoms, aneurysm or venous aneurysm should also be considered as an indication for surgical intervention.

The optimal management of IAVFs has not yet been established due to very few reported cases. As we previously commented, the most common iatrogenic AVFs are femoral AVFs in vascular surgery, the treatment options of which are much more varied compared with IAVFs, including conservative management through compression therapy, open repair, and endovascular repair. The choice of surgical procedures for IAVFs was determined primarily through understanding of the anatomy. Conservative management through compression therapy is often unsuitable for IAVFs. Furthermore, open repair may bring about larger surgical trauma and more complications. Thus, endovascular approach might be more appropriate for patients with IAVFs.

For most patients with IAVFs, relieving the symptoms and inhibiting the progression of fistulas to fatal complications such as severe heart failure and aneurysm rupture were the most realistic goals. After that, what taken into consideration was the radical treatment of AVFs. All the patients in this study were performed endovascular embolization using mainly two different surgical methods. First, transarterial embolization plus stent implantation was performed for IAVFs involving common iliac vessels. As long as possible, placement of a covered stent was preferred to occlude the fistulas. Embolization was not be suitable for common IAVFs with the important role of common iliac artery in blood supply to pelvic organs and lower extremities. However, for internal IAVFs, it is always difficult to perform stent anchoring because of many branches and tortuosity of related vessels close to the fistulas. Second, transarterial embolization was the most frequent and convenient to be performed, which was applied in 50% (8/16) patients with IAVFs. As mentioned above, for patients present with severe cardiac sufficiency, especially congenital IAVFs, it is preferable to give priority to achieving symptom remission. In our experience, for acquired internal patients with IAVFs without symptoms of compression, transarterial embolization could be chosen to be the first attempt. If postembolic angiography showed complete obliteration of the targeting arteries and relief of symptoms after operations, we can stop further intervention and follow-up. If not, repetitive transarterial or stage II transvenous embolization should be taken into consideration. Based on this principle, five patients were treated with stage II transvenous embolization and received a satisfactory clinical result. In addition, two patients present as urinary frequency were performed with simultaneous transarterial and transvenous embolization. The development of compression symptoms suggested that the aneurysm or venous aneurysm was formed and becoming larger due to high-flow blood, which had compressed the surrounding tissues or organs, so stage I two-approach embolization was performed and correspondingly obtained a clinical and technical success. On one hand, the major risk associated with transcatheter embolization of IAVFs is coils migrating to arteries related to the fistulas and next to the lung, resulting in parenchymal or extremity ischemia, or pulmonary embolism, which was not found in this case series (27). On the other hand, except controllable complications, mainly low-grade fever, all the patients were safe in the perioperative period. In the follow-up of 15 well-treated patients with IAVFs, only one patient reported exacerbation of heart failure because of recurrence of primary disease, leiomyosarcoma.

This case study has several limitations to consider when interpreting the findings. First, this was a retrospective study from a single center and the sample size was relatively small. Second, some of the clinical data in the hospital recording system were incomplete and there were no data of open surgery for IAVFs. Thus, a multicenter study with larger sample is needed to evaluate the outcome of endovascular management of IAVFs.

## Conclusion

Iliac arteriovenous fistulas are rare and the clinical manifestations of which is diverse and confusing, so careful physical examination and medical history inquiring are needed for initial diagnosis. In this case series, the number of female and iatrogenic IAVFs were highest because of history of uterine surgery, especially hysterectomy. The primary goal of surgery for IAVFs was relief of symptoms and slowing progression and relatively individualized endovascular procedure was revealed to be a safe and effective method of relieving symptoms though this study.

## Data Availability Statement

The raw data supporting the conclusions of this article will be made available by the authors, without undue reservation.

## Ethics Statement

Written informed consent was obtained from the individual(s) for the publication of any potentially identifiable images or data included in this article.

## Author Contributions

LJ and GG are responsible for the data acquisition, analysis, and manuscript drafting and revising. ZL and YC are responsible for data collection and manuscript revising. WY, BL, and CL are responsible for manuscript revising. YZ is responsible for study design, manuscript revising, and final approval of publish. All authors contributed to the article and approved the submitted version.

## Funding

This study was supported by grants from the Major Research Program of Natural Science Foundation of China (51890894), the Natural Science Foundation of China (81770481 and 82070492) and the Chinese Academy of Medical Sciences, innovation Fund for Medical Sciences (CIFMS 2017-I2M-1-008).

## Conflict of Interest

The authors declare that the research was conducted in the absence of any commercial or financial relationships that could be construed as a potential conflict of interest.

## Publisher's Note

All claims expressed in this article are solely those of the authors and do not necessarily represent those of their affiliated organizations, or those of the publisher, the editors and the reviewers. Any product that may be evaluated in this article, or claim that may be made by its manufacturer, is not guaranteed or endorsed by the publisher.
